# NMDA receptors in neurodegenerative diseases: mechanisms and emerging therapeutic strategies

**DOI:** 10.3389/fnagi.2025.1604378

**Published:** 2025-07-24

**Authors:** Keyi Zhang, Ming Wen, Xinyue Nan, Shuaizhu Zhao, Hao Li, Yanping Ai, Houze Zhu

**Affiliations:** ^1^Innovation Center for Brain Medical Sciences, The Ministry of Education of the People’s Republic of China, Huazhong University of Science and Technology, Wuhan, China; ^2^Department of Pathophysiology, School of Basic Medicine, Tongji Medical College, Huazhong University of Science and Technology, Wuhan, China; ^3^Department of Neurosurgery, Wuhan Hankou Hospital, Hankou Hospital Affiliated to Wuhan University of Science and Technology, Wuhan, China; ^4^Department of Neurology, Wuhan Hankou Hospital, Wuhan, China; ^5^Department of Physiology, School of Basic Medicine, Huazhong University of Science and Technology, Wuhan, China

**Keywords:** NMDA receptors (N-methyl-D-aspartate receptors), neurodegenerative disease, excitotoxicity, glutamate, Alzheimer’s disease (AD), Huntington’s disease (HD), Parkinson’s disease (PD), amyotrophic lateral sclerosis (ALS)

## Abstract

NMDA receptors (NMDARs) are widely distributed throughout the central nervous system (CNS) and play pivotal roles in normal physiological processes such as synaptic plasticity, learning, and memory. Substantial evidence indicates that NMDAR dysfunction, particularly excessive calcium influx, critically contributes to the pathogenesis of major neurodegenerative diseases, including Alzheimer’s disease (AD), Parkinson’s disease (PD), Huntington’s disease (HD), and amyotrophic lateral sclerosis (ALS). Dysregulated glutamatergic signaling synergizes with pathological protein aggregation (e.g., Aβ, *α*-synuclein, mutant huntingtin) to drive neuronal loss. We systematically delineate NMDAR-related mechanisms underlying neurodegeneration, highlighting spatial-specific roles (e.g., synaptic NMDAR-mediated neuroprotection versus extrasynaptic NMDAR-mediated excitotoxicity) and crosstalk with mitochondrial dysfunction and oxidative stress. We critically evaluate current therapeutic strategies targeting NMDARs, including subunit-selective modulators, downstream effector modulation, and glutamate transporter modulation designed to restore NMDAR homeostasis. Consequently, NMDARs and their modulators represent promising therapeutic targets for these refractory conditions. This review comprehensively summarizes current research on the involvement of NMDARs and the glutamatergic system in neurodegenerative diseases. Furthermore, we discuss the clinical application of NMDAR-targeting agents and explore emerging therapeutic strategies focused on modulating NMDAR-related pathways. This article aims to provide a reference for elucidating the molecular mechanisms underlying these neurodegenerative disorders and to highlight potential avenues for future drug development.

## 1 Introduction

Glutamatergic neurotransmission, primarily mediated by N-methyl-D-aspartate receptors (NMDARs), underpins synaptic plasticity, learning, memory and other critical physiological functions (Bannerman et al., 2014; Paoletti et al., 2013; Morris, 2013). However, this critical signaling pathway exhibits a profound duality: its exquisite calcium permeability, essential for physiological processes like long-term potentiation (LTP) (Lüscher and Malenka, 2012), concurrently renders neurons vulnerable to pathological cascades (Kodis et al., 2018). Crucially, NMDAR dysregulation is now recognized not merely as a consequence but as a central driver of the progressive neuronal dysfunction and loss in major neurodegerative diseases, including amyotrophic lateral sclerosis (ALS) (Paul and de Belleroche, 2014; Spalloni et al., 2013), Parkinson’s disease (PD) (Xu et al., 2012; Picconi et al., 2012), Huntington’s disease (HD) (Sepers and Raymond, 2014; Fernandes and Raymond, 2009) and Alzheimer’s disease (AD) (Xu et al., 2012; Wang and Reddy, 2017; Babaei, 2021).

While the neurotoxic potential of excessive NMDAR activation, termed “excitotoxicity,” has been a milestone concept since its first description by Olney in the 1970s (Rothman and Olney, 1987), contemporary research is rapidly dismantling simplistic views, revealing context-dependent signaling outcomes. This complexity simultaneously illuminates novel therapeutic avenues.

The frontier of neurodegeneration research has moved decisively beyond a monolithic view of the NMDARs. Key advances have demonstrated that functional consequences hinge on dynamic interactions among: subunit composition (GluN2A versus GluN2B), subcellular localization (synaptic versus extrasynaptic), developmental stage, neuronal subtype, and associated proteins including scaffolding proteins and signaling effectors (Paoletti et al., 2013; Lohmann and Kessels, 2014; Gladding and Raymond, 2011; Sanz-Clemente et al., 2013). The oversimplified dichotomy attributing neuroprotection exclusively to GluN2A-containing receptors and toxicity to GluN2B-containing receptors has evolved into a more nuanced understanding paradigm. No NMDAR subunit is intrinsically “good” or “bad”; their roles are context-dependent (Hardingham and Bading, 2003). Synaptic NMDARs – often enriched in GluN2A subunits in mature neurons, typically activate pro-survival pathways supporting neuronal plasticity and survival. In contrast, extrasynaptic pools, which frequently contain GluN2B, preferentially couple to mitochondrial dysfunction and oxidative stress when chronically overactivated (such as pathological glutamate spillover) (Hardingham and Bading, 2010). Subunits with restricted expression (such as GluN2D and GluN3A) contribute uniquely to disease-specific vulnerabilities in AD and PD (Crawley et al., 2022; Mellone et al., 2019; Swanger et al., 2015). Developmental and pathological reprogramming further complicates this landscape: GluN2B dominance is vital in neurodevelopment but exacerbates excitotoxicity in mature degenerating neurons. Conversely, AD induces pathological GluN2A internalization and GluN2B surface accumulation, favoring excitotoxic signaling.

Pathologically, neurodegenerative processes actively corrupt this finely tuned NMDAR signaling system. Several universal mechanisms are shared by different neurodegenerative diseases by which NMDAR dysfunction propagates neurodegeneration: (1) excitotoxic calcium overload (Dong et al., 2009; Lau and Tymianski, 2010); (2) synaptic/extrasynaptic receptor imbalance (Hardingham and Bading, 2010; Parsons and Raymond, 2014), (3) mitochondrial dysfunction and oxidative stress (Lin and Beal, 2006; Islam, 2017), and (4) proteinopathy-induced receptor mislocalization (Aβ in AD; *α*-synuclein in PD) (Wang et al., 2013; Durante et al., 2019). Understanding these convergent pathways is key to developing broad-spectrum therapeutic strategies.

The limited disease-modifying efficacy of broad antagonists such as memantine (an uncompetitive, low-affinity, open-channel blocker that preferentially blocks the extrasynaptic NMDAR) (Johnson and Kotermanski, 2006) underscores the futility of non-selective blockade and the imperative for precision targeting. Current strategies focus on: subunit-selective modulators, including GluN2B-selective negative modulators (such as ifenprodil and its derivatives) (Egunlusi and Joubert, 2024) or GluN2A-selective positive modulators (which are less investigated) (Hanson et al., 2020), aiming to restore subunit balance. Location-biased interventions strive to selectively inhibit pathological extrasynaptic NMDAR signaling while sparing crucial synaptic function. Novel strategies such as modulation of NMDAR upstream kinases and phosphatases (such as PKC) and downstream effectors (such as DAPK), dissociation of NMDA receptor complex are also emerging and under active investigation.

This review synthesizes the latest mechanistic insights into multifaceted NMDAR dysregulation in neurodegeneration. We critically evaluate subunit-specific, localization-dependent, and context-governed signaling in disease models and human pathology. Emphasis is placed on translating this complexity into emerging therapeutics that restore physiological homeostasis or selectively block pathological cascades, moving beyond crude receptor inhibition.

## 2 NMDAR composition and spatial distribution

**Figure 1:**
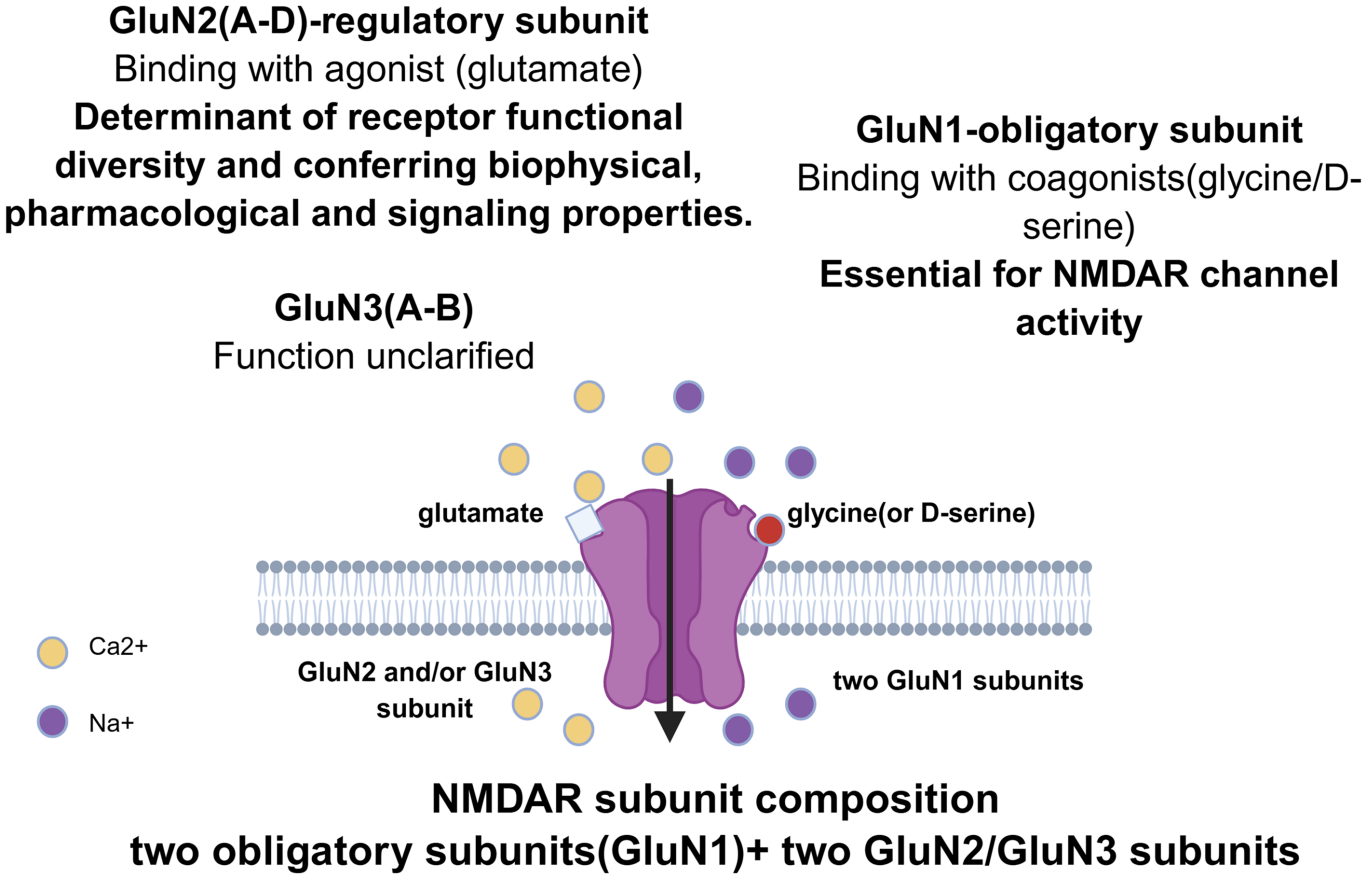
Subunit composition of NMDA receptors. This schematic illustrates the heterotetrameric organization of the NMDARs. Structurally, functional NMDARs are obligate heterotetramers, typically assembled as dimeric pairs of GluN1 subunits and GluN2 subunits. The GluN1 subunit is essential for channel activity, binds the coagonists glycine or D-serine, and exhibits functional diversity through alternative splicing. The regulatory GluN2 subunit, which binds the primary agonist glutamate, confers distinct biophysical properties, pharmacological profiles, and signaling capabilities to the receptor complex. While the precise roles of GluN3 subunits are less defined, emerging evidence indicates their incorporation forms non-canonical NMDARs characterized by reduced calcium permeability and insensitivity to voltage-dependent magnesium block, suggesting unique contributions to neuropathological mechanisms and potential therapeutic targeting.

Structurally, functional NMDARs are obligate heterotetramers, typically assembled as dimeric pairs of glycine-binding GluN1 subunits (the obligatory co-agonist site) and glutamate-binding GluN2 subunits (the agonist site) (Cull-Candy et al., 2001; Hansen et al., 2018) (Figure 1). The GluN1 subunit (encoded by GRIN1) is essential for channel activity and is ubiquitous in all NMDARs (Tu and Kuo, 2015; Chou et al., 2024). The critical functional diversity is conferred primarily from the incorporation of GluN2 subunits (GluN2A-D, encoded by GRIN2A-D, respectively), each conferring distinct biophysical, pharmacological, and signaling properties to the receptor complex (Wyllie et al., 2013; Paoletti, 2011). GluN2A and GluN2B predominate in the forebrain, particularly in specific regions such as the hippocampus and cortex. GluN2C and GluN2D are more restricted, expressed notably in the cerebellum, thalamus, and during early development (Monyer et al., 1994; Watanabe et al., 1994; Köhr, 2006). Additionally, GluN3 subunits (GluN3A-B, encoded by GRIN3A-B), which are not a component of most natural NMDARs, can incorporate into complexes alongside GluN1 and GluN2, forming non-canonical glycine-activated receptors characterized by low calcium permeability and insensitive to magnesium block (Henson et al., 2010; Chatterton et al., 2002). This combinatorial assembly generates a vast array of receptor subtypes with tailored properties.

The spatial distribution of NMDARs within neurons is highly organized and functionally critical. Crucially, NMDARs exhibit a distinct synaptic versus extrasynaptic localization, a dichotomy with profound implications for neuronal signaling and survival (Gladding and Raymond, 2011; Parsons and Raymond, 2014). Synaptic NMDARs, anchored by scaffolding proteins like the PSD-95 family to the postsynaptic density (PSD) (Chen et al., 2015; Niethammer et al., 1996), are activated by vesicular glutamate release into the synaptic cleft. They are key mediators of physiological processes, including the induction of long-term potentiation (LTP) and long-term depression (LTD), the foundation of learning and memory (Lüscher and Malenka, 2012; Bliss and Collingridge, 1993). In contrast, extrasynaptic NMDARs, localized to the plasma membrane outside the PSD such as the dendritic shaft and soma, are often associated with different scaffolds proteins (SAP102 and SAP97) or adhesion molecules (cadherin and catenin) (Petralia et al., 2010; Groc et al., 2009; Li et al., 2011), are primarily activated under pathological conditions involving excessive glutamate spillover or impaired astrocytic glutamate reuptake.

Critically, the location of NMDAR also determines the downstream transcriptional consequences (Bading, 2017). Synaptic NMDAR activation promotes phosphorylation of the transcription factor cAMP response element-binding protein (CREB), driving expression of pro-survival, anti-apoptotic genes such as the brain-derived neurotrophic factor (BDNF) (Greer and Greenberg, 2008) Conversely, extrasynaptic NMDARs induce CREB shut-off pathway while concurrently activating pro-death signaling pathways (Hardingham et al., 2002).

Collectively, this intricate molecular architecture, defined by subunit composition, post-translational modifications, and precise subcellular positioning, establishes the NMDAR as a pivotal regulator of normal brain function. Consequently, disruptions in subunit expression, trafficking, synaptic localization, or downstream signaling cascades contribute profoundly to the pathogenesis of diverse neurodegenerative diseases.

## 3 NMDAR in neuronal impairment: excitotoxicity and beyond

### 3.1 NMDAR-induced excitotoxicity

Neuronal responses to NMDA receptor activity follow a bell-shaped curve where neuronal survival peaks at physiological activation levels but declines under both hypoactive and hyperactive states (Hardingham and Bading, 2010). Hypoactivation of NMDAR has been recognized as a crucial driver in the progression and manifestation of age-related cognitive decline through inhibiting NMDAR-induced LTP and synaptic plasticity and other diseases such as schizophrenia (Nakazawa and Sapkota, 2020; Lindsley et al., 2006; Dong et al., 2023). This paradigm, first established by Hardingham and Bading, arises from spatiotemporal segregation of receptor subtypes. Under physiological conditions, NMDA receptors are briefly activated by a saturating (~1 mM) concentration of glutamate to conduct synaptic transmission (Vyklicky et al., 2014). While under conditions of hypoactivation, NMDAR hypofunction leads to drastic alterations in calcium influx and cellular signalling, impairing receptor transport to the postsynaptic membrane. Conversely, hyperactivation, caused by pathological glutamate spillover (μM concentrations of glutamate) according to different types of pathology or impaired receptor internalization, triggers downstream excitotoxic cascades including ROS production, mitochrondrial dysfunction and eventually neuronal death (Papadia et al., 2005).

**Figure 2:**
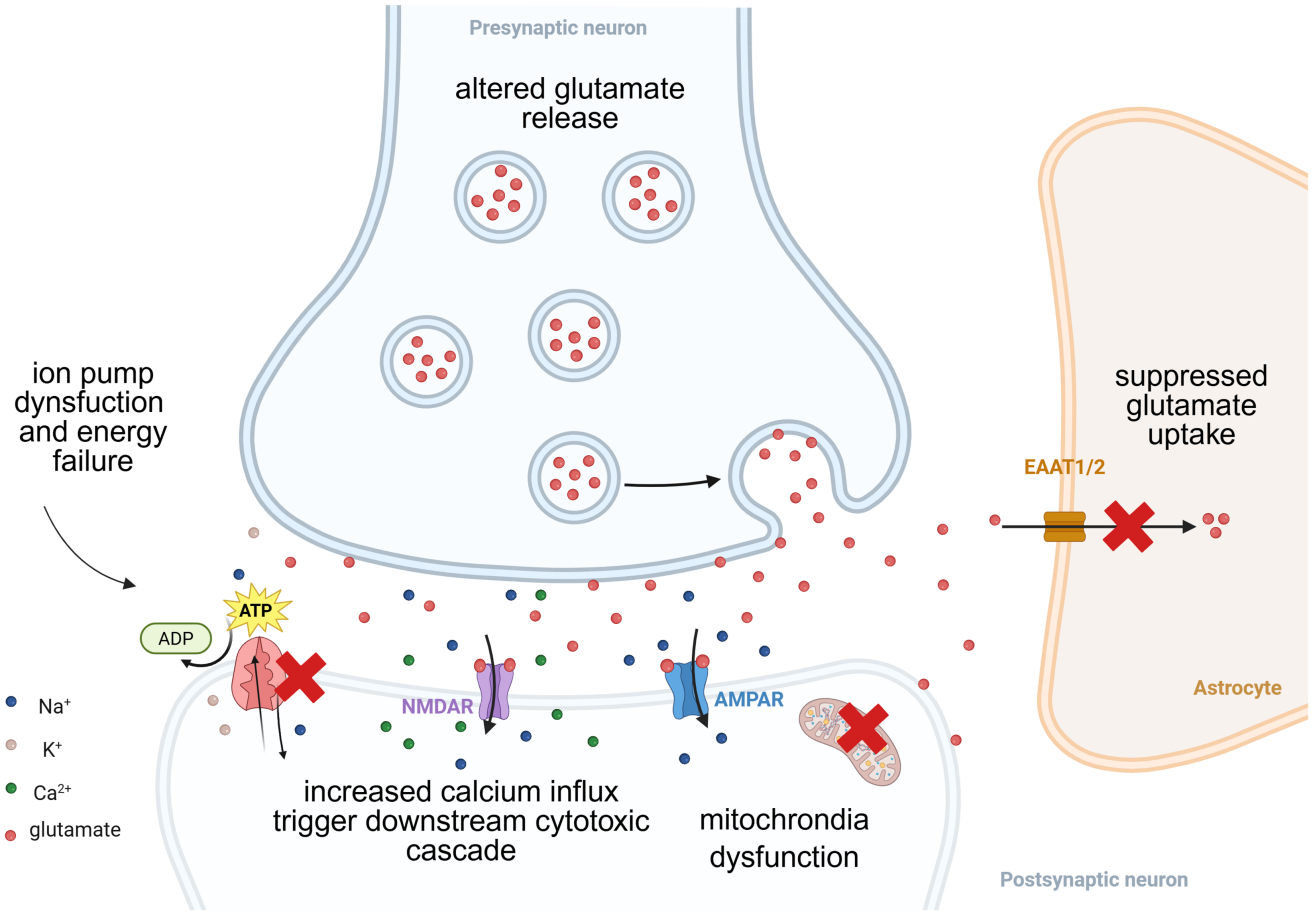
NMDAR-mediated excitotoxic cascade. This schematic illustrates the core sequence of molecular events driving glutamate excitotoxicity: The glutamate reuptake by EAATs fundamentally relies on the transmembrane sodium gradient established and maintained by ATP-dependent Na^+^/K^+^-ATPase activity. Pathological conditions of ischemia, mitochondrial dysfunction, or oxidative stress, impair ATP production, inhibit ion pumps, can lead to the collapse of the ion gradient. Consequently, astrocytic EAATs fail to clear synaptic glutamate. Persistent glutamate accumulation caused sustained NMDAR overactivation, triggering pathological calcium influx that initiates downstream neurotoxic cascades, including further mitochondrial dysfunction and neuronal death.

Excitotoxicity, first described by Olney in the 1970s (Rothman and Olney, 1987), is a critical neurodegenerative mechanism wherein excessive NMDAR activation triggers neuronal death through calcium overload and downstream cytotoxic cascades (Rothman and Olney, 1995) (see Figure 2). This cascade initiates with disrupted glutamate homeostasis. Under physiological conditions, extracellular glutamate concentrations are regulated by excitatory amino acid transporters (EAATs), predominantly EAAT1/2 on astrocytes. The glutamate reuptake mechanism of EAATs fundamentally relies on the transmembrane sodium gradient established and maintained by ATP-fueled Na^+^/K^+^-ATPase activity (Alleva et al., 2020; Alleva et al., 2022). This indirect but absolute energy dependence creates a critical vulnerability point: when under conditions of ischemia, mitochondrial dysfunction, or oxidative stress, which impair ATP production and inhibit the normal function of ion pumps, can lead to the collapse of the ion gradient. Consequently, EAAT-mediated glutamate clearance fails, allowing glutamate to persistently activate postsynaptic NMDARs (Andersen et al., 2021; Murphy-Royal et al., 2017; Mahmoud et al., 2019).

Overactivation of NMDARs further promote calcium influx, initiating downstream neurotoxic cascades: Intracellular calcium surge overwhelms mitochondrial buffering capacity, inducing mitochondrial membrane depolarization, halting ATP synthesis, and reactive oxygen species (ROS) explosion (Neves et al., 2023; Rego and Oliveira, 2003; Szydlowska and Tymianski, 2010). Concurrently, calcium-dependent enzymes initiate destructive processes: calpains degrade cytoskeletal proteins and activate pro-apoptotic Bcl-2 family members (Raynaud and Marcilhac, 2006; Chan and Mattson, 1999); NO causes inhibition of mitochondrial respiratory chain, rapid glutamate release from both astrocytes and neurons, and subsequent excitotoxic death of the neurons (Dawson et al., 1991; Brown and Bal-Price, 2003); phospholipase A2 (PLA_2_) promotes arachidonic acid (AA) release, fueling inflammatory cascades (Sun et al., 2004).

These pathways converge via necrosis, apoptosis, or dysregulated autophagy, constituting a final common pathway in neurodegeneration.

### 3.2 Beyond excitotoxicity: NMDAR as an amplifier of neuronal damage

While the etiology of neurodegenerative diseases remains incompletely understood, emerging evidence implicates defects in energy metabolism and oxidative damage as key co-pathogenic mechanisms beyond excitotoxicity (Beal, 1995; Tripathi et al., 2020; Stark and Bazan, 2011). Involvement of oxidative damage and mitochondrial dysfunction has been suggested as a common feature shared by multiple neurodegenerative diseases (Cenini et al., 2019; Reed, 2011).

Oxidative stress is suggested to be involved in the etiology of both brain aging and neurodegenerative diseases such as AD and PD. (Puspita et al., 2017; Malinski, 2007) NMDAR overactivation leads to excessive calcium influx, which not only triggers excitotoxic cell death but also initiates a cascade of oxidative events. Elevated intracellular calcium binds with calmodulin and activates neuronal nitric oxide synthase (nNOS), to convert l-arginine to citrulline and nitric oxide (NO). NO can function as a messenger molecule in the CNS under physiological conditions, however, when generated in excessive amounts, NO can be neurotoxic (Dawson and Dawson, 1996). NO can be scavenged in a rapid reaction with superoxide (O_2_^−^) to generate peroxynitrite (ONOO^−^). ONOO^−^ is a potent oxidant and the primary component of nitroxidative stress (Malinski, 2007). These free radicals further impair mitochondrial function, creating a vicious cycle of oxidative damage (Duncan and Heales, 2005; Ghasemi et al., 2018). Notably, mitochondrial dysfunction exacerbates ROS production (Murphy, 2009), which may establish a feedforward loop that accelerates neurodegeneration.

Lipid peroxidation, as a critical downstream consequence, directly links oxidative stress to NMDAR regulation and participates in AD, HD, and PD pathophysiology (Reed, 2011). ROS attack polyunsaturated fatty acids (PUFAs) in neuronal membranes to generate highly reactive aldehydes such as 4-hydroxynonenal (4-HNE) and malondialdehyde (MDA) (Gaschler and Stockwell, 2017). These lipid peroxidation products can impair glutamate transport through HHE modification of EAAT2 (Lovell et al., 2012), reducing glutamate clearance and further potentiating NMDAR overactivation.

## 4 Flux-independent NMDAR signaling

Beyond its canonical role as an ionotropic receptor mediating fast excitatory synaptic transmission, emerging evidence highlights the significance of flux-independent (non-canonical or metabotropic-like) NMDAR signaling in neurodegenerative diseases. Several studies have indicated that flux-independent NMDARs mediate LTD, cell membrane molecular dynamics, pH sensing, and synaptic depression induced by amyloid-*β* (Aβ) oligomers (de Oca and Balderas, 2018; Park et al., 2022). Tamburri et al. (2013) demonstrated that oligomeric Aβ induces rapid synaptic depression in hippocampal neurons of slices through a mechanism independent of ion influx but dependent on synaptic NMDAR activaion. Kessels et al. further established that Aβ-induced synaptic depression requires GluN2B-containing NMDARs, as evidenced by blockade with the competitive antagonist D-2-Amino-5-phosphonopentanoic acid (D-APV) (a non-selective GluN2 antagonist) but not by the open-channel blocker MK-801 or glycine-site antagonist 7 chloro-kynurenate (7CK) (Kessels et al., 2013). However, Nabavi et al. reported a contradictory result that 7CK failed to block LTD, which was suspected to be caused by subtle methodological difference (Nabavi et al., 2014). Collectively, these findings support the involvement of flux-independent NMDAR signaling in synaptic dysregulation during neurodegeneration. While this emerging paradigm holds significant therapeutic promise, important limitations remain: the precise molecular mechanisms underlying flux-independent NMDAR signaling are still incompletely characterized, and like most new knowledge, flux-independent NMDARs has been controversial, as contradictory findings exist. These questions are currently under active investigation.

## 5 NMDAR in neurodegenerative diseases

### 5.1 NMDAR and amyotrophic lateral sclerosis (ALS)

Amyotrophic lateral sclerosis (ALS) is a devastating neurodegenerative disease characterized by the progressive loss of upper and lower motor neurons, leading to muscle atrophy, paralysis, and ultimately respiratory failure (Hardiman et al., 2017; Feldman et al., 2022). Although its etiology remains incompletely defined, growing evidence implicates glutamate-mediated excitotoxicity as a key contributor to motor neuron degeneration (Paul and de Belleroche, 2014; Spalloni et al., 2013; Heath and Shaw, 2002).

Motor neurons are particularly vulnerable to NMDAR-mediated excitotoxicity due to their low calcium-buffering capacity and abundant NMDAR expression (Van Den Bosch et al., 2006). Preclinical studies and patient-derived motor neurons have demonstrated that NMDAR overactivation results in mitochondrial dysfunction, oxidative stress, and activation of apoptotic pathways, ultimately culminating in motor neuron death (Paul and de Belleroche, 2014; Heath and Shaw, 2002; Menzies et al., 2002; Catania et al., 2001; Paul et al., 2014).

Interrupted glutamate homeostasis further contributes to excitotoxicity. Studies have revealed elevated levels of glutamate and aspartate in the cerebrospinal fluid of ALS patients (Rothstein et al., 1990). This accumulation is strongly linked to the selective loss or dysfunction of the major astrocytic glutamate transporter, EAAT2, observed in the motor cortex and spinal cord of ALS patients and in transgenic ALS mouse (SOD1 mutant) models (Rothstein et al., 1995; Howland et al., 2002). Critically, the reduced EAAT2 expression is induced by dysregulated NF-κB signaling, which represses EAAT2 expression (Frakes et al., 2014; Crosio et al., 2011). Paradoxically, NF-κB is also required for both activation and repression of the EAAT2 promoter, which positioning it as a context-dependent regulator (Kim et al., 2011). For example, N-myc and NF-κB are required for TNF-*α*-mediated transcriptional repression of EAAT2. On the contrary, NF-κB also mediates EGF-, TGF-α-, and cAMP-induced EAAT2 promoter activation (Su et al., 2003; Sitcheran et al., 2005). Besides its effects on glutamate transporters, NF-κB may also contribute to ALS pathogenesis by induction of pro-inflammatory gene expression (Källstig et al., 2021). Consequently, excessive synaptic glutamate persistently activates NMDARs. Oxidative stress may also participate in ALS pathogenesis through oxidative stress-mediated protein injury, lipid peroxidation, and DNA and RNA oxidation have been observed in ALS patients (Singh et al., 2019).

Beyond glutamate, pathological accumulation of the NMDAR co-agonist D-serine driven by impaired degradation such as the DAO R199W mutation shifts its role from physiological modulator to neurotoxic effector (Sasabe et al., 2012). Excess D-serine drives NMDAR overstimulation that exacerbates neurodegeneration through two convergent pathways: direct potentiation of excitotoxic calcium influx via extrasynaptic NMDARs, and indirect induction of non-excitotoxic death mechanisms including autophagic flux blockade and intrinsic apoptosis (Mitchell et al., 2010; Kondori et al., 2018).

ALS-associated genetic mutations further implicate NMDAR dysfunction. For example, the C9ORF72 mutation, the most common genetic cause of ALS/FTD (Pang and Hu, 2021), contributes to neurodegeneration through disrupting the surface expression, transport, and recycling of NMDARs. Studies using induced motor neurons (iMNs) derived from C9ORF72 ALS/FTD patients revealed elevated expression of the essential GluN1 subunit on neurites and dendritic spines (Burk and Pasterkamp, 2019; Shi et al., 2018). This increase facilitates more frequent calcium influx, thereby exacerbating excitotoxicity.

**Figure 3:**
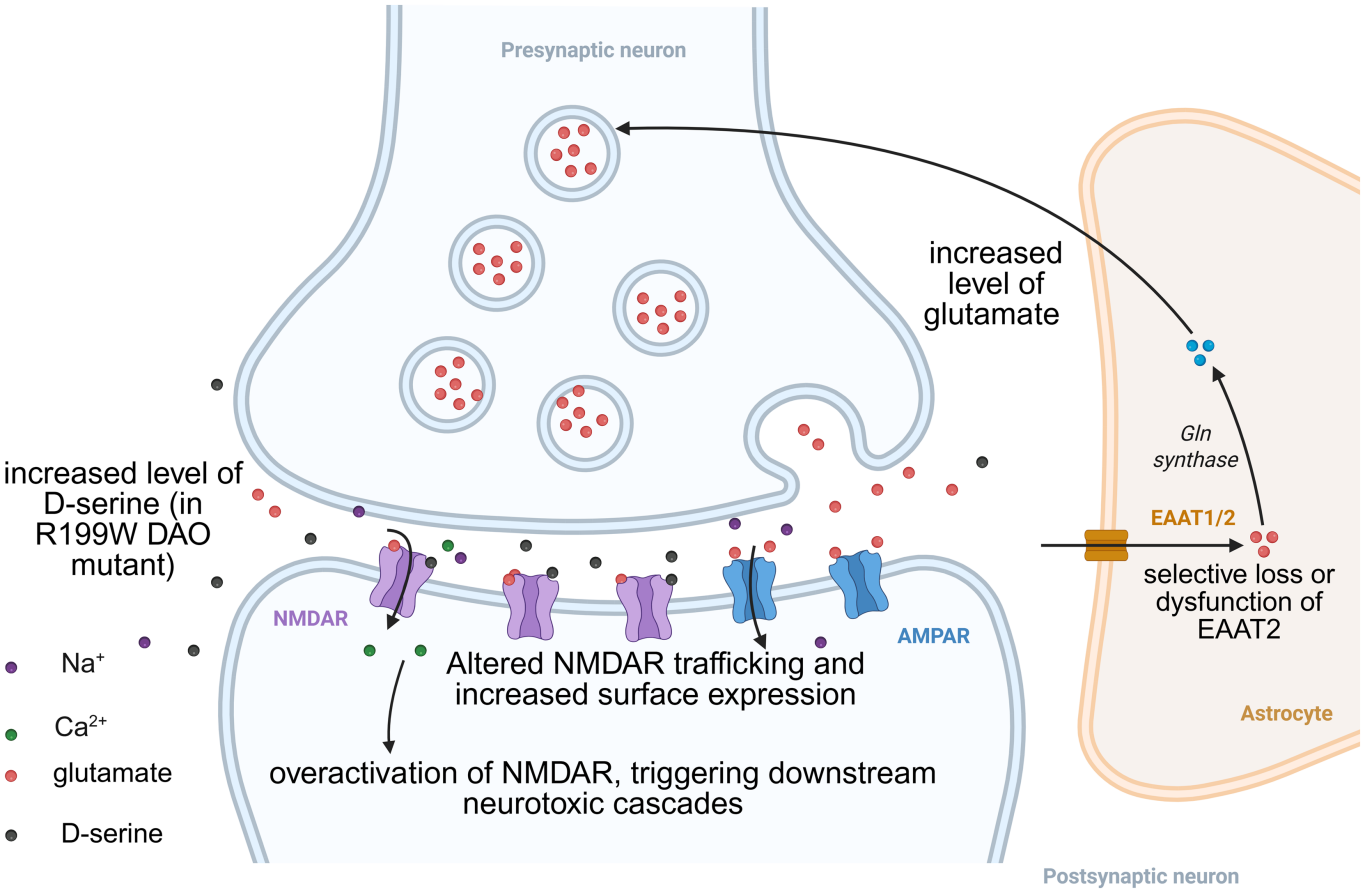
NMDAR dysregulation in amyotrophic lateral sclerosis (ALS). This schematic illustrates the key mechanisms driving excitotoxicity in ALS. Interrupted glutamate homeostasis is a key promoter in the pathogenesis of ALS, primarily due to selective loss or dysfunction of EAAT2. Pathologically elevated levels of the NMDAR coagonist D-serine, resulting from impaired degradation due to the DAO R199W mutation, further promote NMDAR activation. Altered NMDAR trafficking and increased surface expression, characterized by elevated GluN1 subunit density in neurons harboring C9ORF72 mutations, provide an additional substrate for overactivation. These astrocytic and neuronal defects collectively sustain NMDAR overactivation, facilitating pathological calcium influx and exacerbating excitotoxic injury to motor neurons.

The central role of NMDAR-mediated excitotoxicity makes NMDAR a compelling therapeutic target in ALS. However, the complexity of ALS pathogenesis (Figure 3) has hindered the development of effective disease-modifying treatments. The only FDA-approved drug, riluzole, has modest efficacy, and can only extend the average survival time by 3 months and cannot reverse motor neuron damage (Miller et al., 2012). Direct NMDAR antagonists (such as memantine) have yielded limited clinical success, largely due to their disruptive effects on essential physiological NMDAR functions in synaptic plasticity and cognition, leading to unacceptable side effects. While preclinical studies in SOD1 mutant mice demonstrate that memantine treatment delays disease progression and improves motor neuron survival, likely through inhibition of spinal cord NMDA receptors (Wang and Zhang, 2005; Joo et al., 2007), these findings have not translated to clinical benefit in ALS patients. Notably, several clinical trials evaluating memantine in sporadic ALS showed that though memantine is well-tolerated in ALS patients, no significant effects on disease progression or survival time were found (de Carvalho et al., 2010; Bhai et al., 2025). Thus, while memantine remains a valuable tool for investigating NMDAR-mediated mechanisms in ALS models, current evidence does not support its therapeutic use in patients.

Consequently, research is shifting towards more refined approaches, such as subunit-selective NMDAR antagonists or modulators targeting allosteric sites. Additionally, combination therapies targeting both upstream triggers (such as enhancing glutamate uptake) or downstream effectors of NMDAR overactivation (such as anti-apoptotic agents, antioxidants), even gene therapies, alongside selective NMDAR modulation hold promise for providing broader neuroprotection for ALS patients (Jiang et al., 2022).

### 5.2 NMDAR and Parkinson’s disease (PD)

Parkinson’s disease (PD) is a progressive neurodegenerative disorder characterized by the loss of dopaminergic neurons in the substantia nigra pars compacta (SNc) and the formation of Lewy bodies. This pathology leads to core motor symptoms including bradykinesia, rigidity, and resting tremor, as well as non-motor manifestations such as cognitive impairment and psychiatric disturbances (Kalia and Lang, 2015; Hayes, 2019; Melzer and Monyer, 2020).

Chronic NMDAR overactivation is a key mechanism of dopaminergic neuron degeneration (Beal, 1998). Basal ganglia circuit imbalance, specifically disinhibition of the subthalamic nucleus (STN) due to striatal dopamine depletion, drives excessive glutamate release onto substantia nigra pars compacta (SNc) neurons, resulting in sustained NMDAR stimulation and downstream neurotoxic events (Dunah et al., 2000; Rodriguez et al., 1998). This excitotoxic cascade is compounded by two intrinsic vulnerabilities of SNc neurons: low expression of calcium-buffering proteins (notably calbindin-D28K) (Liang et al., 1996; Sulzer and Surmeier, 2013) and high surface density of GluN2B-containing NMDARs (Suárez et al., 2010; Jones and Gibb, 2005). Besides, misfolded *α*-synuclein oligomers exacerbate NMDAR hyperactivation by inducing astrocytic glutamate release (Trudler et al., 2021). These interactions jointly trap NMDARs in a hyperactive state, exacerbating calcium influx and overwhelming mitochondrial calcium buffering capacity.

Beyond excitotoxicity, NMDAR dysregulation in PD also contributes to impaired synaptic plasticity and the non-motor symptoms of the disease, such as cognitive impairment and depression. An unbalanced GluN2A/GluN2B subunit ratio of the striatal synaptic NMDAR is thought to be a crucial determinant in the regulation of motor behaviour and synaptic plasticity in PD (Mellone et al., 2015). Pathological alterations include: (1) an imbalanced GluN2A/GluN2B ratio with selective depletion of GluN2B-containing receptors; (2) reduced phosphorylation of GluN1 and GluN2B; (3) dopamine D1 receptor-dependent redistribution of NMDARs between synaptic and postsynaptic sites (Landwehrmeyer et al., 1995; Paillé et al., 2010; Zhang and Chergui, 2015; Dunah and Standaert, 2001). These changes collectively impair synaptic function and may mediate adverse effects of dopaminergic therapy such as the levodopa-induced dyskinesia (LID) (Zhang et al., 2023).

Therapeutically, NMDAR antagonists show dual promise: they protect SNc neurons in preclinical models and ameliorate motor complications. Amantadine, an antagonist of NMDAR as an adjuvant to levodopa therapy, has been found to significantly ameliorate motor complications in PD and supports the idea that NMDAR hyperfunction contributes to levodopa-associated motor complications (Papa and Chase, 1996; Greenamyre and O'Brien, 1991; Metman et al., 1998). Memantine exhibits more modest efficacy and lacks amatadine’s anti-dyskinetic activity (Merello et al., 1999). The second generation of adamantane-based drugs is being designed, seeking to improve the clinical efficacy (Dembitsky et al., 2020). Given the dual role of NMDARs in motor and non-motor symptoms, future therapies may need to adopt a multifaceted approach, targeting specific receptor subtypes or brain regions to address the diverse manifestations of PD.

### 5.3 NMDAR and Huntington’s disease (HD)

Huntington’s disease (HD) is an autosomal dominant disorder caused by CAG trinucleotide expansions in the HTT gene that results in polyglutamine (polyQ)-expanded mutant huntingtin (mHTT) protein. This mutation drives progressive striatal degeneration largely through mHTT-induced NMDAR dysregulation, manifesting as motor dysfunction, cognitive decline, and psychiatric disturbances (McColgan and Tabrizi, 2018; Walker, 2007).

The selective vulnerability of striatal medium spiny neurons (MSNs) stems from their high GluN2B-NMDAR expression and intense corticostriatal glutamatergic input, rendering them particularly sensitive to mHTT-induced alterations in NMDAR trafficking, localization, and signaling (Landwehrmeyer et al., 1995; Li et al., 2003; Cepeda et al., 2007).

The pathogenic cascade begins with mHTT disrupting postsynaptic organization: mHTT exhibits reduced binding to PSD-95 compared to wild-type HTT (Sun et al., 2001). Yet mHTT paradoxically enhances PSD-95/GluN2B interactions in HD models, which may be linked to increased extrasynaptic NMDAR mislocation in HD (Fan et al., 2009; Milnerwood et al., 2010). This shift toward extrasynaptic NMDAR dominance creates a permissive environment for excitotoxicity, which has been suggested as a major player in HD pathogenesis (Fan and Raymond, 2007; Raymond et al., 2011).

Concurrently, elevated GluN3A subunit expression in HD striatum accelerates afferent synapse loss onto medium spiny neurons (MSNs). Notably, suppressing GluN3A in the YAC128 HD mouse model corrects NMDAR hyperexcitability, rescues synapses, ameliorates motor and cognitive deficits, and reduces striatal atrophy (Marco et al., 2013; Wesseling and Pérez-Otaño, 2015).

Therapeutic strategies targeting NMDAR dysfunction in HD are evolving to address the complexity of receptor dysregulation, with a growing emphasis on subunit-selective modulation and the restoration of synaptic-extrasynaptic NMDAR balance. GluN2B-selective antagonists such as memantine have shown considerable promise in preclinical models. In a small pilot trial in HD patients, 20 mg of memantine daily intake can significantly improve motor symptoms (Ondo et al., 2007). Besides, the application of neurotrophic factors, autophagy regulators, stem cells, and genetic therapies are also under investigation for HD treatment (Kim et al., 2021).

### 5.4 NMDAR and Alzheimer’s disease (AD)

**Figure 4:**
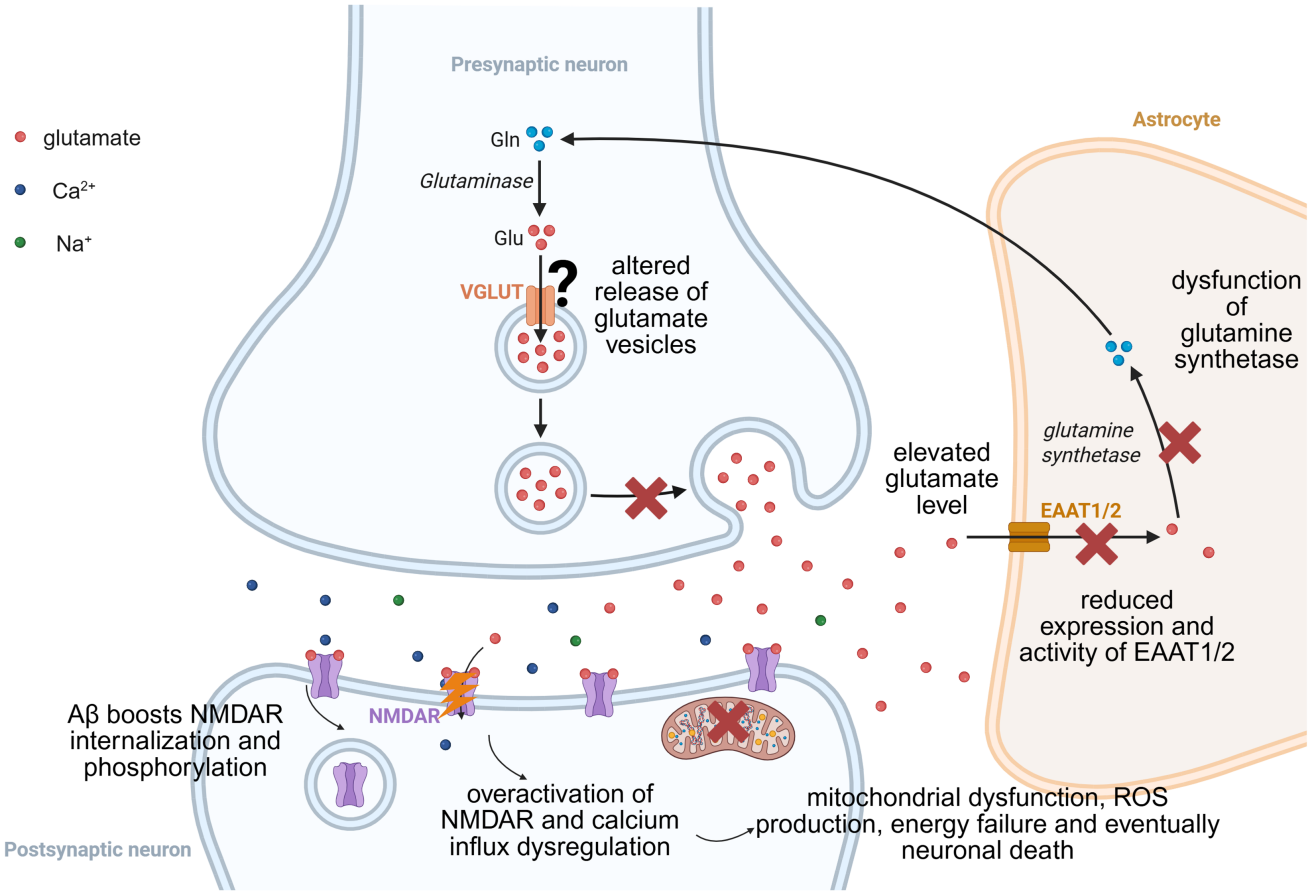
Aβ-induced excitotoxic cascade via NMDAR dysregulation in Alzheimer’s disease. This schematic illustrates the Aβ-triggered pathological cycle: Aβ oligomers drive presynaptic glutamate spillover through impaired glutamate transporter expression, decreased glutamine synthetase activity, and altered vesicular glutamate release. Postsynaptic NMDARs undergo Aβ-induced phosphorylation and internalization, leading to pathological overactivation and calcium influx dysregulation. Resultant calcium overload triggers mitochondrial dysfunction, reactive oxygen species (ROS) production, energy failure and eventually neuronal death. This self-amplifying cascade culminates in neuronal edema, synaptic loss, and neurodegeneration.

Alzheimer’s disease (AD), the most prevalent neurodegenerative cause of dementia, is defined by progressive cognitive decline alongside neuropathological hallmarks including amyloid-*β* (Aβ) plaques and neurofibrillary tau tangles (Lane et al., 2018; Scheltens et al., 2021). Central to AD pathogenesis is NMDAR dysregulation, manifesting through excitotoxicity, synaptic failure, and bidirectional interactions with Aβ/tau pathology (Wang and Reddy, 2017; Raïch et al., 2024; Escamilla et al., 2024) (see Figure 4).

Aβ oligomers were described to accumulate in the AD patient brain, or *in vitro* in human cortex neuronal cultures, at GluN2B-containing synapses. Aβ oligomers bind to cellular prion protein (PrPc) and metabotropic glutamate receptor 5 (mGluR5), forming a complex that promotes phosphorylation of the GluN2B subunits. This triggers initial NMDAR surface accumulation followed by internalization, driving calcium overload, mitochondrial dysfunction, calpain activation, and dendritic spine loss (Um et al., 2013; Um et al., 2012; De Felice et al., 2007). Concurrently, Aβ disrupts glutamate homeostasis by mislocalizing astrocytic EAAT2 transporters and impairing glutamine synthetase (GS) activity, elevating extracellular glutamate and sustaining NMDAR stimulation (Scimemi et al., 2013; Aksenov et al., 1995). Aβ additionally induces pathological NMDAR subunit switching (GluN2B to GluN2A) and alters vesicular glutamate release via vGluT1 and/or vGluT2 downregulation (Kessels et al., 2013; Rodriguez-Perdigon et al., 2016; Mi et al., 2023). Additionally, reduced activity and expression of GS have been reported in both patient tissue and animal models, contributing to the glutamate homeostasis disruption (Kulijewicz-Nawrot et al., 2013; Robinson, 2000). Tau pathology amplifies this dysfunction when hyperphosphorylated tau impairs synaptic NMDAR trafficking while promoting receptor internalization (Hoover et al., 2010).

The distribution of synaptic versus extrasynaptic NMDARs has emerged as a key profile in neurodegenerative diseases including AD. In AD, extrasynaptic NMDARs oppose synaptic NMDARs by triggering CREB (a master regulator of synaptic plasticity) shut-off and promoting mitochondrial dysfunction and neuronal death (Hardingham et al., 2002; Wang et al., 2004; Esposito et al., 2013). CREB is a crucial molecular factor for learning and memory and its downregulation is assumed to result in cognitive deficits in AD (Rosa and Fahnestock, 2015; Bartolotti et al., 2016). Critically, Aβ downregulates CREB phosphorylation, and suppresses downstream BDNF expression (Amidfar et al., 2020; Garzon and Fahnestock, 2007).

Mitochondrial-ROS dysregulation converges on NMDAR pathology: Reduced PGC-1α levels in AD impair ROS detoxification and reduce mitochondrial density, diminishing neuronal resilience to excitotoxic stress (Sweeney and Song, 2016; Qin et al., 2009; Wareski et al., 2009; Cui et al., 2006).

Mechanistically, NF-κB signaling also plays a crucial role in AD pathogenesis by regulating different molecules responsible for promoting the morbidities associated with AD (Sun et al., 2022). NF-κB induces the expression of *β*-secretase, resulting in the formation of amyloid fibrils, which consequently aggregate into amyloid plaques (Cole and Vassar, 2007). Similarly, Aβ oligomers can in turn stimulate NF-κB activation in neurons and glial cells, forming a vicious cycle (Snow and Albensi, 2016).

Therapeutic strategies targeting NMDAR dysfunction in AD have evolved from simple receptor blockade to more nuanced approaches aimed at restoring physiological receptor function. Memantine remains the only FDA-approved drug targeting NMDARs for AD treatment, however its efficacy remains modest (Xia et al., 2010; Glasgow et al., 2017; Reisberg et al., 2003). Current research mainly focuses on subunit-selective modulators and spatially-targeted agents like NitroMemantine, a selective extrasynaptic NMDAR antagonist developed on the basis of memantine, which targets specific localized NMDARs, is of great potential for AD treatment.

## 6 NMDA receptor as a target for treating neurodegenerative diseases

NMDARs represent pivotal therapeutic targets for neurodegenerative diseases, governing excitotoxicity, synaptic dysfunction, and neuronal survival. Their dualistic nature: physiological activation supporting cognition versus pathological overstimulation driving degeneration, demands precisely calibrated interventions. Current therapeutic strategies prioritize activity-dependent modulation over complete receptor blockade. Subunit-selective agents now dominate therapeutic innovation: GluN2B-selective antagonists (e.g., ifenprodil and its derivatives) preferentially target neurodegenerative extrasynaptic receptors without impairing cognition in AD and HD models (Ugale et al., 2024), while GluN2A-positive allosteric modulators (PAMs) counteract synaptic depletion in late-stage disease (Yukawa et al., 2023).

Novel therapeutic strategies further expand the landscape: (1) Targeting kinases/phosphatases upstream of NMDARs (such as PKC activator bryostatin-1) or effectors downstream (such as DAPK1/NR2B uncoupler) (Zhang et al., 2020). Bryostatin-1 demonstrates efficacy in AD trials by reducing Aβ, promoting synaptogenesis, and suppressing oxidative stress, with favorable safety profiles enabling clinical application (Hongpaisan et al., 2011; Tian et al., 2023). In contrast, administration of a peptide NR2B^CT1292–1,304^ to uncouple the activated DAPK1 from the NMDA receptor complex protects against brain damage, which indicates that targeting DAPK1-NMDA receptor interaction can be considered as a practical strategy (Wang et al., 2017); (2) Receptor complex dissociation: Small molecules like ZL006 (uncoupling NMDAR/PSD-95) selectively block neurotoxic NO signaling without impairing physiological receptor function (Tao et al., 2020); (3) Neural circuit rebalancing (Ghatak et al., 2021), Restoring excitatory/inhibitory (E/I) imbalances caused by extrasynaptic NMDAR hyperactivity in autism and AD (Vico Varela et al., 2019; Schuch et al., 2016).

Despite robust preclinical evidence for NMDAR modulation in neurodegeneration, clinical translation has been hampered by intersecting pharmacological and biological barriers. Blood–brain barrier (BBB) penetration remains a primary bottleneck (Egunlusi and Joubert, 2024). Species divergence in receptor biology further complicates the clinical translation, rendering compounds optimized for murine receptors ineffective (such as the different effect of memantine in ALS on animal model and patients mentioned before). Crucially, achieving subunit-or localization-specific drug delivery in human patients is also a key barrier underlying the current NMDAR-based therapeutics. Emerging therapies aiming to overcome these hurdles include novel drug delivery systems such as lipid nanoparticles, loaded with riluzole (Bondì et al., 2010) and dopamine (Ortega Martínez et al., 2024), have shown promising results in increasing drug bioavailability in the CNS for ALS and PD treatment.

The future of NMDAR-targeted therapy lies in personalized combinatorial approaches: integration of subunit-selective drugs, neuroprotective agents, and disease-modifying treatments tailored to disease stage and specific pathology of each neurodegenerative disorder. By addressing both the excitotoxic mechanisms and the broader cellular context of NMDAR dysfunction, these strategies hold significant potential to slow or even halt neurodegeneration.

## 7 Discussion

The multifaceted role of NMDA receptors in neurodegenerative diseases has emerged as a central paradigm in understanding both the pathogenesis and potential treatment strategies for these neurodegenerative diseases. Our synthesis of current evidence reveals that NMDAR dysfunction operates through a complex, interconnected network of mechanisms that vary across different neurodegenerative disorders while sharing common pathological themes. At the core of this dysregulation lies the delicate balance between synaptic and extrasynaptic NMDAR signaling, a delicate balance that becomes profoundly disrupted in disease states. The consequences of this imbalance manifest through multiple converging pathways: excitotoxic calcium overload, oxidative stress, mitochondrial dysfunction, impaired synaptic plasticity, and maladaptive transcriptional changes.

Though the application of memantine and other NMDAR antagonists in different neurodegenerative diseases has shown certain potential, however, the efficacy of existing NMDAR antagonists is still limited and often result in significant side effects, such as euphoria, psychotic symptoms and increased blood pressure, indicating gaps in our current understanding of the diseases and the complexity of NMDAR functions (Muir, 2006). Recent advances in structural biology and receptor pharmacology have enabled the design of compounds with unprecedented specificity for particular NMDAR subtypes and locations. GluN2B-selective antagonists such as ifenprodil and its deriavtives (Gogas, 2006) represent a significant step forward by preferentially targeting receptors implicated in neurodegeneration while sparing those essential for cognitive function. Several recent studies have also investigated the potential of novel GluN2A-targeting positive allosteric modulators such as AGE-718 and 6-methylpyridin-2-one (Yukawa et al., 2023; Beckley et al., 2024), which also offers a complementary strategy to bolster synaptic resilience. Together, these approaches aim to restore the physiological balance between neuroprotective and neurotoxic NMDAR signaling.

Beyond direct receptor modulation, innovative strategies targeting downstream effectors (such as DAPK1) or upstream kinases/phosphatases (such as PKC) offer complementary value. Small molecules that disrupt the GluN2B-PSD95-nNOS complex such as ZL006 demonstrate how specific protein–protein interactions can be targeted to block neurotoxic signaling while preserving physiological receptor function.

The future of NMDAR-targeted therapy lies in precision medicine frameworks, considering the patient-specific genetic and molecular profiles to tailor NMDAR-targeting therapies. Another major frontier is the optimization of drug delivery to overcome the blood–brain barrier while maintaining therapeutic concentrations in relevant brain regions. The emergence of novel delivery systems, such as nanoparticle carriers holds promise for addressing this challenge. Perhaps most importantly, future therapies must account for the dynamic nature of NMDAR changes throughout disease progression. The receptor alterations that drive early synaptic dysfunction may differ substantially from those mediating late-stage neuronal death, suggesting that optimal interventions may need to evolve with disease progression.

The remarkable progress in understanding NMDAR biology over the past decades has transformed our approach to understand and treat neurodegenerative diseases. From viewing these receptors primarily as mediators of excitotoxicity, we now appreciate their roles in diverse pathological processes ranging from protein misfolding to neuroinflammation. This expanded understanding has given rise to a new generation of therapeutic strategies that seek not just to block excessive NMDAR activity, but to restore the delicate balance of synaptic and extrasynaptic signaling. As we continue to unravel the complexities of NMDAR regulation in health and disease, the prospect of developing truly disease-modifying treatments grows increasingly tangible. The path forward will require continued collaboration across disciplines, from structural biology to clinical neurology. By building on the foundations laid by current research and embracing the challenges that remain, we may finally be able to translate our knowledge of NMDAR mechanisms into transformative therapies for neurodegenerative diseases.

## Glossary

NMDAR: N-methyl-D-aspartate receptor
ALS: Amyotrophic Lateral Sclerosis
PD: Parkinson’s Disease
HD: Huntington’s Disease
AD: Alzheimer’s Disease
LTP: Long-term Potentiation
LTD: Long-term Depression
PSD: Postsynaptic Density
AMPAR: Amino-3-hydroxy-5-methyl-4-isoxazolepropionic acid receptor
CNS: Central Nervous System
EAAT2: Excitatory Amino Acid Transporter 2
CREB: cAMP Response Element-binding Protein
BDNF: Brain-derived neurotrophic factor
ROS: Reactive Oxygen Species
nNOS: neuronal Nitric Oxide Synthase
PLA2: Phospholipase A2
PUFAs: Polyunsaturated Fatty Acids
AA: Arachidonic Acid
4-HNE: 4-hydroxynonenal
MDA: Malondialdehyde
D-APV: D-2-Amino-5-phosphonopentanoic acid
7CK: 7 chloro-kynurenate (7CK)
SNc: Substantia Nigra Pars Compacta
STN: Subthalamic Nucleus
LID: Levodopa-induced Dyskinesia
DAPK1: Death-associated Protein Kinase 1
NF-κB: Nuclear Factor Kappa-Light-Chain-Enhancer of Activated B Cells
HTT: huntingtin
mHTT: mutant Huntingtin
MSNs: Medium Spiny Neurons
PrPc: Cellular Prion Protein
mGluR5: Metabotropic Glutamate Receptor 5
A*β*: Amyloid-β
GS: Glutamine Synthetase
